# Bootstrap-Optimised Regularised Image Reconstruction for Emission Tomography

**DOI:** 10.1109/TMI.2019.2956878

**Published:** 2020-01-14

**Authors:** Andrew J. Reader, Sam Ellis

**Affiliations:** School of Biomedical Engineering and Imaging SciencesKing’s College London, King’s Health Partners, St Thomas’ HospitalLondonSE1 7EHU.K.

**Keywords:** Image reconstruction, inverse problems, emission tomography, regularisation, hyperparameter selection, bootstrap methods

## Abstract

In emission tomography, iterative image reconstruction from noisy measured data usually results in noisy images, and so regularisation is often used to compensate for noise. However, in practice, an appropriate, automatic and precise specification of the strength of regularisation for image reconstruction from a given noisy measured dataset remains unresolved. Existing approaches are either empirical approximations with no guarantee of generalisation, or else are computationally intensive cross-validation methods requiring full reconstructions for a limited set of preselected regularisation strengths. In contrast, we propose a novel methodology embedded within iterative image reconstruction, using one or more bootstrapped replicates of the measured data for precise optimisation of the regularisation. The approach uses a conventional unregularised iterative update of a current image estimate from the noisy measured data, and then also uses the bootstrap replicate to obtain a bootstrap update of the current image estimate. The method then seeks the regularisation hyperparameters which, when applied to the bootstrap update of the image, lead to a best fit of the regularised bootstrap update to the conventional measured data update. This corresponds to estimating the degree of regularisation needed in order to map the noisy update to a model of the mean of an ensemble of noisy updates. For a given regularised objective function (e.g. penalised likelihood), no hyperparameter selection or tuning is required. The method is demonstrated for positron emission tomography (PET) data at different noise levels, and delivers near-optimal reconstructions (in terms of reconstruction error) without any knowledge of the ground truth, nor any form of training data.

## I. Introduction

Iterative image reconstruction and parameter estimation methods are often compromised by the noise present in the raw measured data, and so regularisation of some form is usually required. Maximum likelihood (ML) parameter estimation, such as image reconstruction for emission tomography (ET) data, is an important example of this [1], [2] among many. While ML has been a theoretically motivated approach to ET image reconstruction and has benefits in terms of accurate noise modelling of the raw data and use of an accurate system model, it is hampered by overfitting of the noisy data. To avoid overfitting, there have been three main approaches proposed in the iterative ET image reconstruction literature. The first approach is simply to stop the iterative process early before excessive levels of noise manifest in the images. However, while noise is successfully limited, the problem of selecting how many iterations to use arises, as well as spatially-variant convergence, which can give misleading results and a lack of definition as to what has been optimised during the reconstruction. A second approach is to apply post-reconstruction smoothing to remove noise, but again one needs to choose the level of smoothing, and there is once again a lack of definition as to what has been optimised. Thirdly, regularisation can be included in the objective function in order to compensate for noise in a theoretically-justified manner. This can be achieved through maximum *a posteriori* (MAP) (also known as penalised likelihood) methods, which introduce an extra term in the reconstruction objective function to counteract image noise [1], and also via reparameterisation methods that use alternative basis functions (e.g. [3]–[5]). In both cases though, the user is still required to select hyperparameters to control the level of regularisation, either in the form of penalty strengths for MAP methods, or through the number of iterations and the basis-function design parameters for the basis function approaches.

A further weakness of traditional noise-compensation and regularisation methods is that the optimum level of regularisation is dependent on the noise level within the data, i.e. noisier datasets require higher levels of regularisation. In some contexts, such as ET imaging, the level of noise is highly variable due to practical limitations. Using historical or empirical regularisation levels in these contexts can therefore lead to sub-optimal parameter estimates, reducing the amount of useful information available from a given acquired dataset.

There has therefore been a long-standing need for an image reconstruction framework which a) does not require the user to select hyperparameters, b) does not overfit the data (resulting in erratic, noisy reconstructed images), and c) does not underfit the data (resulting in overly smooth, biased reconstructions lacking in detail).

Previous work targeting this need includes cross-validation, which is an effective method for model selection, but its use in ET image reconstruction thus far has been very limited. The first main attempt was overly simple—whereby cross-validation was merely used as a stopping criterion [6]. This leaves the results with spatially-variant convergence issues characteristic of early termination methods, and furthermore provides no assurance of optimality of the reconstructions. Hence this first attempt at including cross-validation in ET reconstruction not only lacked any form of model selection (which is the usual goal of cross validation) but also lacked any ability to account for spatially-variant convergence. Another proposed method for hyperparameter selection is the use of L-curves [7]. However, this requires many experimental reconstructions with different choices of regularisation hyperparameter followed by a selection of the corner of the L-curve—amounting to a large computational burden of many reconstructions and a degree of user specification in finding the corner of the L-curve. More recently, Zhang *et al* [8] proposed a more advanced method based on cross-validation, however this method also requires complete reconstructions for each user preselected choice of hyperparameter, resulting in a computationally demanding and imprecise grid search of the hyperparameter.

A related area of research is spatially-adaptive regularisation, which encourages certain characteristics of the reconstructed image to be more homogeneous throughout the field-of-view by taking into account the spatially-variant trade-off between resolution and noise that occurs in regularised tomographic image reconstruction. For example, previous research has attempted to produce spatially-invariant image resolution when using a penalised image reconstruction [9]. Using a similar methodology, other work has focused on providing spatially-invariant noise levels [10], [11]. However, despite adjusting the level of regularisation spatially to influence image characteristics, these methods still require the overall level of regularisation to be provided by the user [10], resulting in the possibility of over- or under-regularisation if sub-optimal levels are chosen.

The novel proposal of this work is to use one (or optionally more) bootstrap replicates of the measured data, generated just once before reconstruction, in order to find a precise hyperparameter for regularisation automatically “on-the-fly” during the iterative image reconstruction process. The advantages are that the reconstruction method a) only requires user specification of the type of penalty or prior, b) obviates the need to select any values or ranges for the hyperparameters, c) obviates the need to carry out multiple complete reconstructions for a limited preselected set of candidate hyperparameter values. Furthermore, the method not only provides precise hyperparameter values, but is also more computationally efficient than previous exhaustive methods for hyperparameter selection, since it just performs one iterative image reconstruction procedure, requiring update steps based on one or more fixed bootstrapped replicates of the data. Note that in this present work we are only tackling the problem of regularisation for noise compensation, rather than for underdetermined systems, which would remain for potential future work.

The structure of this paper is as follows. In Section II, the theory behind the proposed method is presented, followed in Section III by a description of example implementations for ET image reconstruction. In Sections IV and V the proposed method is applied to a 2D simulation study and real 3D data respectively, with comparison to conventional reconstruction methods. The results and implications of these experiments are discussed in Section VI, with conclusions in Section VII.

## II. Theory

Image reconstruction aims to estimate a vector of object-representation parameters $\boldsymbol {\theta }$ (e.g. voxel intensities for an image) given a vector of measured data $\boldsymbol {m}$, which for ET can be regarded as a list of counts for various measurement elements (e.g. sinogram bins). The MAP objective function for image reconstruction can be written as

\begin{equation*} \Phi ^{\textrm {MAP}}(\boldsymbol {\theta }) = \Phi ^{\textrm {ML}}(\boldsymbol {\theta }; \boldsymbol {m}) - \beta R(\boldsymbol {\theta }) \tag{1}\end{equation*}

where $\Phi ^{\textrm {ML}}(\boldsymbol {\theta }; \boldsymbol {m})$ is the likelihood of the parameter vector $\boldsymbol {\theta }$ given the measured data $\boldsymbol {m}$, $R(\cdot)$ is some regularising penalty function in terms of the parameters of interest and $\beta $ is a hyperparameter that controls the strength of the regularisation. In general, more than one regularisation hyperparameter can be used, and the method proposed here can be applied to such cases, but for simplicity this work will focus on the most common case of just one hyperparameter.

The strength of the regularisation in (1) is a very important consideration. If $\beta $ is too high, the reconstructed image is over-regularised, over-compensating for the noise present in the data, resulting in overly-biased images lacking detail. In contrast, if $\beta $ is too low, the noise reduction is insufficient and images can be difficult to interpret with potentially misleading features. This work proposes a novel, bootstrap-based method for finding $\beta $ with precision during the iterative reconstruction process, based on modelling the mean of an ensemble of noisy reconstructions, as will be described below. The method has no recourse to any previously learned or empirically determined selection mechanism, and does not need multiple reconstructions to be performed for different hyperparameters.

For iterative image reconstruction methods, the image update for seeking the maximum of (1) is:

\begin{equation*} \boldsymbol {\theta }^{(k+1)} = U(\boldsymbol {\theta }^{(k)}; \boldsymbol {m}) \tag{2}\end{equation*}

where $\boldsymbol {\theta }^{(k)}$ denotes the image estimate at iteration $k$, and $U$ is an update formula whose form depends on the objective function and optimisation algorithm. A conventional choice for $U$ in ET is the expectation maximisation (EM) algorithm [12], for progression towards the ML estimate.

For the general MAP image reconstruction case with a fixed regularisation strength of $\beta $, the update step can be written as:

\begin{equation*} \boldsymbol {\theta }^{(k+1)} = U_{\beta }(\boldsymbol {\theta }^{(k)}; \boldsymbol {m}). \tag{3}\end{equation*}

Using this definition, the unregularised image update based on fidelity to the measured data only is

\begin{equation*} \boldsymbol {\theta }_{\textrm {meas}}^{(k+1)} = U_{\beta =0}(\boldsymbol {\theta }^{(k)}; \boldsymbol {m}). \tag{4}\end{equation*}

For many algorithms, the regularised image update can be rewritten as a function of the unregularised image update, given a fixed value of $\beta $. In effect, this is a noise-compensation update:

\begin{equation*} \boldsymbol {\theta }^{(k+1)} = {F_{\beta }}\!{\left ({\boldsymbol {\theta }_{\textrm {meas}}^{(k+1)}}\right)}. \tag{5}\end{equation*}

Again, the exact form of $F_\beta (\cdot)$ depends on the regulariser and optimisation algorithm used. In this work we require $F_\beta (\cdot)$ to be found from an algorithm which is known to converge to the MAP estimate, for a fixed $\beta $. Below we will show an explicit example for the regularising function considered in this work.

### A. Ensemble-Mean Optimised Regularisation

We first hypothetically consider a large set, or *ensemble*, of independent noisy data realisations, each obtained for exactly the same object in the scanner field of view, and acquired for exactly the same period of time, such that each has approximately the same number of counts as contained in the actual measured data vector $\boldsymbol {m}$. For each of the datasets in this ensemble, it would be possible to find an unregularised update via (4), such as the EM update. We would therefore have a set of unregularised updates, from which the ensemble mean of all noisy updates can be found. Clearly, this mean of all the updates will be noise free as the number of datasets in the ensemble tends to infinity, and therefore (in the context of completely sampled ET data) this mean update would be a highly desirable goal for any iterative reconstruction algorithm.

In the proposed method, we seek to estimate the regularisation hyperparameter $\beta $ such that the regularised update of the parameter vector given by (5) matches the ensemble mean of the set of unregularised updates. Of course, in practice, we only have one measured dataset $\boldsymbol {m}$, and so it is not possible to know the ensemble mean of the set of unregularised updates. Instead, we propose modelling this situation via bootstrapping. Specifically, we model the desired ensemble mean simply by the unregularised update of the single measured dataset (4), and we model the unregularised noisy update by an unregularised update based on a bootstrap resampled replicate of the measured data ($\boldsymbol {m}_{\textrm {boot}}$):

\begin{equation*} \boldsymbol {\theta }_{\textrm {boot}}^{(k+1)} = U_{\beta =0}(\boldsymbol {\theta }^{(k)}; \boldsymbol {m}_{\textrm {boot}}). \tag{6}\end{equation*}

We then seek the value of $\beta $ which will map this model of the unregularised update (obtained from bootstrapped data) to match the model of the ensemble mean (given simply by the unregularised update from the original measured data). This means that at iteration $k$, we find two provisional updates: one update according to (4), and then also an update of the parameter vector using bootstrapped data according to (6).

Hence we propose fitting the bootstrap update of the image given by (6) to the conventional unregularised update given by (4) via optimisation of the hyperparameter(s) of the regularisation noise-compensation operator ${F_\beta }{\left ({\cdot }\right)}$. Having found the optimal hyperparameters through this model, these same hyperparameters can be applied to the standard unregularised image update (4), to obtain a noise-compensated iterative update of the image (5). This update is an estimate of the unknown ensemble mean of numerous updates based on independent noisy acquired datasets.

Fitting an update from bootstrapped data to the update found from the measured data can be achieved by optimisation of any of the numerous possible objective functions, to give what this work will refer to as an ensemble-mean objective function (EMOF), $C$. This objective could be, for example, a distance measure to minimise, or a likelihood to maximise, and in this work the former is used. The optimum $\beta $ value at a given iteration in the reconstruction process, denoted $\beta _{\textrm {opt}}^{(k)}$, is given by:

\begin{equation*} \beta _{\textrm {opt}}^{(k)} = \mathop {\mathrm {arg\,min}} _\beta {C}{\left ({\boldsymbol {\theta }_{\textrm {meas}}^{(k+1)},{F_{\beta }}\!{\left ({\boldsymbol {\theta }_{\textrm {boot}}^{(k+1)}}\right)}}\right)}. \tag{7}\end{equation*}

**Fig. 1.**
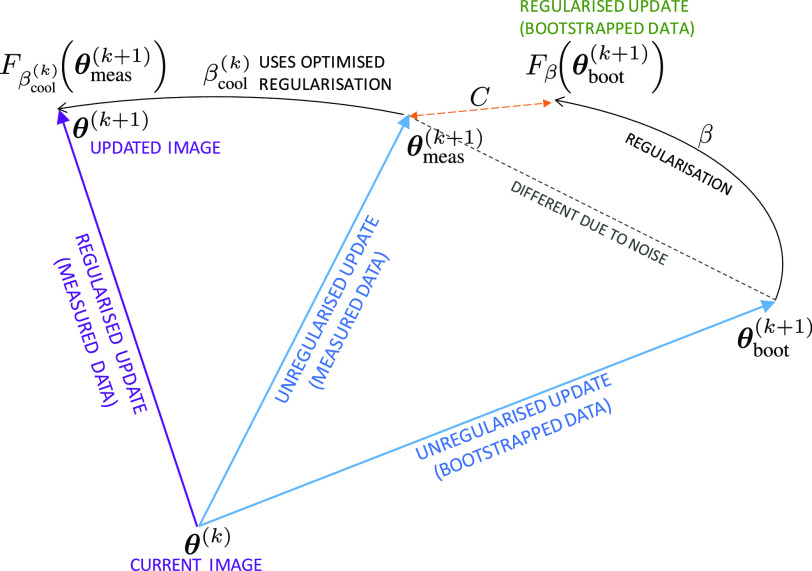
Illustration of the principles of the method. Applying an unregularised update to $\boldsymbol {\theta }^{{({k}}{)}}$ using the measured data gives $\boldsymbol {\theta }_{\text {meas}}^{({k}+{1}{)}}$, and using the bootstrapped data gives $\boldsymbol {\theta }_{\text {boot}}^{({k}+{1}{)}}$. The update from measured data is used as a model of the mean of an ensemble of noisy updates, and the update from bootstrapped data is a model of an example noisy update. The bootstrap-optimised regularisation method therefore seeks a $\beta $ which best fits the bootstrap image to the measured data image, using a given regularisation function ${F}_{\beta }$. Once found, the value can be used in the processing of the measured data update, so as to regularise the update and obtain $\boldsymbol {\theta }^{({k}+{1}{)}}$.

In (7) the goal is to find the optimal hyperparameter $\beta _{\textrm {opt}}$ for the noise-compensation operator ${F_{\beta }}\!\left ({\cdot }\right)$ which when applied to the bootstrap update leads to an image that best fits the conventional update (Figure 1).

There are many possible options for functions to use as the EMOF in (7), including simple $\ell _{p}$-norms or the Kullback-Leibler divergence as is used in nested EM for dynamic PET image reconstruction [13]. In this work we use the $\ell _{2}$-norm for $C$, corresponding to a simple Gaussian log-likelihood, which has the benefit of robustness when values close to zero arise.

### B. Hyperparameter Fitting

In what follows, it will be helpful to consider the spatial frequency content of the updated image estimate $\boldsymbol {\theta }^{(k+1)}$. We will proceed with two postulates. The first is based on the properties of the Fourier transform and the Riemann-Lebesgue lemma: the noise-free component of $\boldsymbol {\theta }^{(k+1)}$ has spatial-frequency representation coefficients which tend to decrease with increasing spatial frequency. The second postulate is that the noise component of $\boldsymbol {\theta }^{(k+1)}$ has spatial-frequency representation coefficients which either remain approximately constant for all frequencies (white noise), or which increase with increasing spatial frequency (arising from the iterative reconstruction operator progressively amplifying the higher spatial frequencies in order to counteract the decaying modulation transfer function of the imaging system, or more generally, its decaying singular value spectrum). With these postulates, the signal to noise ratio (SNR) in the spatial frequency domain therefore decreases with higher spatial frequencies. This is an almost ubiquitous assumption throughout all the ET literature, and is the unfailing experience in practice (e.g. [14]). Now, we can regard the regularisation strength $\beta $ as controlling the roll-off, or even cut-off, in spatial frequencies present in the regularised image update. The purpose of (7) is to find the best $\beta $ that controls this roll-off in spatial frequencies to just the right level, so as to diminish the coefficients for spatial frequencies which are noise dominated. Clearly, it is the noise-dominated spatial frequencies which result in a discrepancy between the unregularised update $\boldsymbol {\theta }_{\textrm {meas}}^{(k+1)}$ and the regularised update ${F_{\beta }}\!{\left ({\boldsymbol {\theta }_{\textrm {boot}}^{(k+1)}}\right)}$. If no noise were present in the measured $\boldsymbol {m}$ (effectively infinite counts), then there would be no discrepancy between the updates $\boldsymbol {\theta }_{\textrm {meas}}^{(k+1)}$ and $\boldsymbol {\theta }_{\textrm {boot}}^{(k+1)}$.

If we try to minimise $C$ in (7) when all spatial frequencies are present in both $\boldsymbol {\theta }_{\textrm {meas}}^{(k+1)}$ and ${F_{\beta }}\!{\left ({\boldsymbol {\theta }_{\textrm {boot}}^{(k+1)}}\right)}$ we can immediately see that only a small, sub-optimal, value of $\beta $ will be found. This is because $\boldsymbol {\theta }_{\textrm {meas}}^{(k+1)}$ will contain all spatial frequencies, including the possibly noise dominated high spatial frequencies. Using this as the target for the EMOF will mean that $\beta $ cannot be as large as it may need to be, as if $\beta $ attenuates the high spatial frequencies in $\boldsymbol {\theta }_{\textrm {boot}}^{(k+1)}$ then these diminished values (potentially close to zero) will be a cause of mismatch with $\boldsymbol {\theta }_{\textrm {meas}}^{(k+1)}$, which retains all its high frequency components.

Therefore it is necessary, initially, to eliminate the higher spatial frequencies from consideration when seeking $\beta $, and only gradually to include them once $\beta $ has first been given opportunity to be as large as is necessary to compensate for noise at the lower spatial frequencies first. Here we suggest two ways of achieving this, and in this work we proceed with the second of the two suggestions. The first proposal is to systematically consider a series of differently smoothed updates, and find the largest value of $\beta $, for any of those smoothing levels, which is required in order to best fit ${F_{\beta }}\!{\left ({\boldsymbol {\theta }_{\textrm {boot}}^{(k+1)}}\right)}$ to $\boldsymbol {\theta }_{\textrm {meas}}^{(k+1)}$. However, this approach will be slow, and requires a search over different smoothing levels. The second proposal is to impose an over-regularisation scheme early on in the series of iterative updates, which is gradually reduced, while constantly searching for, and keeping as a baseline, the maximum value of beta that is found necessary to fit ${F_{\beta }}\!{\left ({\boldsymbol {\theta }_{\textrm {boot}}^{(k+1)}}\right)}$ to $\boldsymbol {\theta }_{\textrm {meas}}^{(k+1)}$.

It is clear that for early iterations of an algorithm which performs simultaneous updates of all pixels/voxels (e.g. the EM algorithm), whereby only the lower spatial frequencies are being recovered, only relatively small values of $\beta _{\textrm {opt}}$ are required, as the SNR is usually at its highest for the lower spatial frequencies. As iterations continue, and as the over-regularisation is reduced in order to allow higher spatial frequencies to be recovered, then larger values of $\beta _{\textrm {opt}}$ are found necessary, as the SNR is poorer for these higher frequencies. Larger $\beta _{\textrm {opt}}$ values are found with increasing iterations and decreasing over-regularisation, but only up to a certain level of over-regularisation. As the level of over-regularisation diminishes, and as more iterations are considered, so more and more high spatial frequencies components are recovered in the reconstructed image update $\boldsymbol {\theta }^{(k+1)}$. Now it is clear, based on the postulate that spectral SNR is a decreasing function of frequency, that as more high frequencies are permitted to be recovered, so the level of noise apparent in the reconstructed image will increase. Just as mentioned earlier, if we try to minimise $C$ in (7) when all these spatial frequencies are present we can immediately see only a small value of $\beta _{\textrm {opt}}$ will be found, as larger values of $\beta $ for $F_{\beta }$ eliminate higher spatial frequencies components from $\boldsymbol {\theta }_{\textrm {boot}}^{(k+1)}$, causing greater discrepancy with $\boldsymbol {\theta }_{\textrm {meas}}^{(k+1)}$, which retains all its high frequency components.

Therefore, with increasing iterations, and with decreasing over-regularisation we eventually observe a decrease in the $\beta _{\textrm {opt}}$, down to a fixed, stable value. The final value of $\beta _{\textrm {opt}}$ corresponds to the best fit of ${F_{\beta }}\!{\left ({\boldsymbol {\theta }_{\textrm {boot}}^{(k+1)}}\right)}$ to $\boldsymbol {\theta }_{\textrm {meas}}^{(k+1)}$ when all spatial frequencies are under consideration, which as explained already, will not be a good fit. So we propose using the largest $\beta _{\textrm {opt}}$ that was found, denoted $\beta _{\textrm {use}}$, as this corresponds to just the right amount of roll-off/cut-off in spatial frequencies before the above phenomenon occurs, whereby inclusion of more spatial frequencies only increases the discrepancy between ${F_{\beta }}\!{\left ({\boldsymbol {\theta }_{\textrm {boot}}^{(k+1)}}\right)}$ and $\boldsymbol {\theta }_{\textrm {meas}}^{(k+1)}$.

\begin{equation*} \beta _{\textrm {use}}^{(k)} = \max \left ({\left \lbrace{ \beta _{\textrm {opt}}^{(1) },\ldots, \beta _{\textrm {opt}}^{(k)}}\right \rbrace }\right). \tag{8}\end{equation*}

This seeks the largest possible $\beta $ which can fit ${F_{\beta }}\!{\left ({\boldsymbol {\theta }_{\textrm {boot}}^{(k+1)}}\right)}$ to $\boldsymbol {\theta }_{\textrm {meas}}^{(k+1)}$, corresponding to the highest possible roll-off/cut-off frequency which can match these two updates.

### C. Over-Regularisation Cooling Schedule

As discussed above, an imposed over-regularisation is initially required to ensure the best $\beta _{\textrm {opt}}$ is found. This over-regularisation needs to be gradually reduced, to allow more spatial frequencies to be considered in the optimisation process. The over-regularisation is given by a factor which is gradually decreased (cooled) with increasing iterations, allowing gradual progression towards the case of finally allowing only the fitted level of regularisation $\beta _{\textrm {use}}$ to be applied. Many cooling schemes are possible, and the one used here is:

\begin{equation*} \beta _{\textrm {cool}}^{(k)} = \beta _{\textrm {use}}^{(k)} + \lambda ^{(k)} \beta _{\textrm {opt}}^{(k)}, \tag{9}\end{equation*}

where $\lambda ^{(k)}$ is the level of over-regularisation at iteration $k$, given by the following decay formula

\begin{equation*} \lambda ^{(k)} = \lambda ^{(0)}{\exp }{\left ({-\frac {k}{N}}\right)}, \tag{10}\end{equation*}

such that $\beta _{\textrm {cool}}$ tends to $\beta _{\textrm {use}}$. Here $\lambda ^{(0)}$ is the initialisation of the over-regularisation factor (referred to as the cooling initialisation) and $N$ is the cooling constant. It is important to note that provided $\lambda ^{(0)}$ and $N$ are both sufficiently large, then they need never be changed or adapted, but can be left as constants for all manner of noise levels under consideration, as will be demonstrated in Section IV.

Note further that while $\beta _{\textrm {cool}}$ can be orders of magnitude greater than $\beta _{\textrm {use}}$ in early iterations, the cooling scheme is designed to ensure that at higher iterations the reconstruction performs iterations with the fixed value of $\beta _{\textrm {use}}$ found by (8). This finally ensures the optimisation of (1) with a fixed $\beta $ value selected during the reconstruction.

### D. Summary

At a given iterative update, after finding $\beta _{\textrm {opt}}$ by (7), updating $\beta _{\textrm {use}}$ by (8), and finding the current $\beta _{\textrm {cool}}$ by (9), the final step of the method is to use $\beta _{\textrm {cool}}$ in the regularised image update:

\begin{equation*} \boldsymbol {\theta }^{(k+1)} = {F_{ \beta _{\textrm {cool}}^{(k)}}}\!{\left ({\boldsymbol {\theta }_{\textrm {meas}}^{(k+1)}}\right)} \tag{11}\end{equation*}

The overall bootstrap-optimised regularised image reconstruction algorithm is summarised in Algorithm 1.

Algorithm 1 **Algorithm~for Bootstrap-Optimised Regularised Iterative Image Reconstruction**
Input: Measured data $\boldsymbol {m}$, unregularised iterative update formula, $U_{\beta =0}(\boldsymbol {\theta }^{(k)}; \boldsymbol {m})$, regularisation/denoising function ${F_{\beta }}\!{\left ({\cdot }\right)}$
Output: Reconstructed image $\boldsymbol {\theta }^{(\textrm {maxIter})}$
*Initialisation*: Set the initial image estimate $\boldsymbol {\theta }^{(0)}$ to a uniform image, the cooling initialisation $\lambda ^{(0)}$, the cooling constant $N$, and the maximum number of iterations maxIter such that maxIter is at least 3–4 times $N$;
1: Generate a bootstrap replicate of the measured sinogram, $\boldsymbol {m}_{\textrm {boot}}$;
2: **for** $k = 1$ to maxIter **do**
3: Calculate the standard image update according to (4);
4: Calculate the bootstrap image update according to (6);
5: Find the bootstrap-optimised regularisation hyperparameter $\beta _{\textrm {opt}}^{(k)}$ according to (7);
6: Find the maximum $\beta _{\textrm {opt}}$ value to give $\beta _{\textrm {use}}^{(k)}$ according to (8);
7: Calculate the current over-regularisation factor, $\lambda ^{(k)}$, according to (10);
8: Calculate $\beta _{\textrm {cool}}^{(k)}$ according to (9);
9: Apply this hyperparameter value to get the next overall image update according to: $\boldsymbol {\theta }^{(k+1)} = {F_{ \beta _{\textrm {cool}}^{(k)}}}\!{\left ({{ \boldsymbol {\theta }}_{\textrm {meas}}^{(k+1)}}\right)}$;
10: **end for**
11: **return** $\boldsymbol {\theta }^{(\textrm {maxIter})}$

## III. Example Implementation

The proposed method should be applicable to any regularised ET image reconstruction or parameter estimation problem for which noise is a concern. Here we present its use for positron emission tomography (PET) for two example regularisation functions: the quadratic penalty with and without guidance information.

In PET, the most common unregularised iterative image reconstruction algorithm is the maximum likelihood expectation maximisation (MLEM) algorithm [12]. This algorithm is obtained by considering the Poisson noise of the measured PET data, and aims to maximise the Poisson log-likelihood of the parameters of interest given the measured data. Given an image estimate $\boldsymbol {\theta }^{(k)}$ and the measured data vector $\boldsymbol {m}$, the MLEM update is written as

\begin{align*} \boldsymbol {\theta }_{\textrm {MLEM}}^{(k+1)} & = U_{\textrm {MLEM}}(\boldsymbol {\theta }^{(k)}; \boldsymbol {m}) \\ &= \frac { \boldsymbol {\theta }^{(k)}}{ \boldsymbol {A}^{T} \boldsymbol {1}} \boldsymbol {A}^{T}\frac { \boldsymbol {m}}{ \boldsymbol {A} \boldsymbol {\theta }^{(k)} + \boldsymbol {b}}\tag{12}\end{align*}

where $\boldsymbol {A}$ is the system matrix, $\boldsymbol {b}$ is an estimate of the mean background (scatters and randoms), and $\boldsymbol {1}$ denotes a vector of ones equal in length to $\boldsymbol {m}$. Multiplication and division of two vectors (where vectors are always identified through use of a lower-case font in bold italics in this work) are taken to be element-wise, whereas for matrix-vector multiplications, conventional notation is preserved. This notation follows the proposal of Barrett *et al* [15].

### A. Generating a Bootstrap Replicate

Depending on whether the measured data are in list-mode or sinogram form, there is more than one way of obtaining a bootstrap replicate $\boldsymbol {m}_{\textrm {boot}}$ of emission tomography data $\boldsymbol {m}$ (e.g. see [16]–[18]). Here we assume a list-mode form, which can easily be generated from binned sinograms as follows: each sinogram bin is examined in turn, and if the bin holds one count or more, then the index of that bin is copied to a list according to the number of counts in the bin. For example, if sinogram bin number $i$ has $n$ counts, then index $i$ is copied $n$ times into the output list. The end result is a list of bin indices with as many entries as there are counts in the sinogram data. This list is then uniformly and randomly sampled from, with replacement, to generate a bootstrapped list of sinogram bin indices. From this, output bootstrapped sinogram data can be formed by just reading each index from the bootstrap list and incrementing the count in the corresponding output sinogram index. For example, sinogram bin index $i$ will occur in our bootstrapped list $n'$ times, resulting in the $i$th bin of $\boldsymbol {m}_{\textrm {boot}}$ containing $n'$ counts.

### B. Bootstrap-Optimised Quadratic Penalty

The proposed method is demonstrated using the well-known example of a quadratic penalty, as used numerous times throughout the ET image reconstruction literature (e.g. [19], [20]). In this case, the regularisation function for (1) is given by

\begin{equation*} R_{\textrm {Q}}(\boldsymbol {\theta }) = \frac {1}{4}\sum _{j=1}^{J}\sum _{l\in \mathcal {N}_{j}}w_{jl}\left ({\theta _{j} - \theta _{l}}\right)^{2} \tag{13}\end{equation*}

where $w_{jl}$ is a weight specifying the strength of regularisation between voxel $j$ and a given voxel $l$ in the neighbourhood of $j$, denoted $\mathcal {N}_{j}$. The quadratic penalty discourages images with neighbourhood differences, as these are assumed to correspond to noise. However, the use of weights allows specification of which pairs of voxels are permitted to be different in value (e.g. edges in the image), by reducing the penalty for differences between such pairs (e.g. [21]).

A robust algorithm for solving this MAP image reconstruction problem in PET is the modified EM algorithm, as proposed by De Pierro [22]. The MAPEM update formula in this case, as rewritten by [23], is:

\begin{equation*} U_{\beta }(\boldsymbol {\theta }^{(k)}; \boldsymbol {m}) = \frac {2 \boldsymbol {\theta }_{\textrm {MLEM}}^{(k+1)}} { \boldsymbol {\xi }_{\beta }^{(k+1)}+\sqrt {\Big (\boldsymbol {\xi }_{\beta }^{(k+1)}\Big)^{2} + 4\beta \boldsymbol {\nu } \boldsymbol {\theta }_{\textrm {MLEM}}^{(k+1)}} }\quad \tag{14}\end{equation*}

where the image $\boldsymbol {\theta }_{\textrm {MLEM}}^{(k+1)}$ is the standard MLEM update as defined in (12), the elements of the vector $\boldsymbol {\nu }$ are given by

\begin{equation*} \nu _{j} = \frac {\sum _{l\in \mathcal {N}_{j}}w_{jl}}{s_{j}}, \tag{15}\end{equation*}

where $\boldsymbol {s} = \boldsymbol {A}^{T} \boldsymbol {1}$, and

\begin{equation*} \boldsymbol {\xi }_\beta ^{(k+1)} = 1 - \beta \boldsymbol {\nu } \boldsymbol {\theta }_{\textrm {reg}}^{(k+1)}, \tag{16}\end{equation*}

where $\boldsymbol {\theta }_{\textrm {reg}}^{(k+1)}$ is a smoothed image given by:

\begin{equation*} \theta _{j,\textrm {reg}}^{(k+1)} = \frac {1}{2\sum _{l\in \mathcal {N}_{j}}w_{jl}}\sum _{l\in \mathcal {N}_{j}}w_{jl}\left ({\theta _{l}^{(k)} + \theta _{j}^{(k)}}\right). \tag{17}\end{equation*}

To adapt the modified EM to include the proposed bootstrap-optimised regularisation we define the unregularised standard and bootstrap image updates as

\begin{align*} \boldsymbol {\theta }_{\textrm {meas}}^{(k+1)}=&U_{\textrm {MLEM}}(\boldsymbol {\theta }^{(k)}; \boldsymbol {m}) \tag{18a}\\ \boldsymbol {\theta }_{\textrm {boot}}^{(k+1)}=&U_{\textrm {MLEM}}(\boldsymbol {\theta }^{(k)}; \boldsymbol {m}_{\textrm {boot}}).\tag{18b}\end{align*}

The denoising operator ${F_{\beta }}\!{\left ({\cdot }\right)}$ is given by (14) so that

\begin{equation*} {F_{\beta }}\!{\left ({\boldsymbol {\theta }_{\textrm {boot}}^{(k+1)}}\right)} = \frac {2 \boldsymbol {\theta }_{\textrm {boot}}^{(k+1)}} { \boldsymbol {\xi }_{\beta }^{(k+1)} + \sqrt {\Big (\boldsymbol {\xi }_{\beta }^{(k+1)}\Big)^{2} + 4\beta \boldsymbol {\nu } \boldsymbol {\theta }_{\textrm {boot}}^{(k+1)}} }. \tag{19}\end{equation*}

$\beta _{\textrm {opt}}^{(k)}$ is the value of $\beta $ which when used in (19) produces the result closest to $\boldsymbol {\theta }_{\textrm {meas}}^{(k+1)}$, as required by the EMOF (7). This is a 1D optimisation problem given by

\begin{equation*} \beta _{\textrm {opt}}^{(k)} = \mathop {\mathrm {arg\,min}} _\beta \left \Vert{ \boldsymbol {\theta }_{\textrm {meas}} ^{(k+1)} - {F_{\beta }}\!{\left ({\boldsymbol {\theta }_{\textrm {boot}}^{(k+1)}}\right)}}\right \Vert _{2}^{2}. \tag{20}\end{equation*}

Given the form of ${F_{\beta }}\!{\left ({\cdot }\right)}$ in (19), it is difficult (perhaps impossible) to find a closed-form solution for $\beta _{\textrm {opt}}^{(k)}$. For this reason we use standard iterative algorithms for this 1D optimisation, described in the relevant sections below. Once $\beta _{\textrm {opt}}^{(k)}$ is found, the values of $\beta _{\textrm {use}}^{(k)}$ and $\beta _{\textrm {cool}}^{(k)}$ are obtained by (8) and (9), and $\beta _{\textrm {cool}}^{(k)}$ is applied to $\boldsymbol {\theta }_{\textrm {meas}}^{(k+1)}$ to obtain $\boldsymbol {\theta }^{(k+1)}$.

## IV. 2D Simulation Studies

To evaluate the performance of the proposed bootstrap-optimised reconstruction method, 2D simulation studies using PET image reconstruction were conducted. These studies were designed to test the method with a range of noise levels, and compare results with conventional image reconstruction. Data generation and reconstruction were performed in MATLAB 2017a (The MathWorks, Natick, MA, USA), with the Radon transform and its adjoint used for projection and backprojection respectively.

### A. Experimental Methods

#### 1) Data Simulation:

A 2D digital ground truth PET radiotracer distribution based on the BigBrain phantom [24], [25] was generated in a $643 \times 643$ pixel grid corresponding to a pixel side length of $400\mu \text{m}$. The distribution was designed to mimic the uptake of [^18^F]fluorodeoxyglucose (FDG). Simulated data were generated by first introducing blurring in the object space by convolution with a Gaussian kernel of 4.5mm FWHM to model the intrinsic limited spatial resolution in PET, and then projecting the blurred phantom into a sinogram containing 180 azimuthal angles and 185 radial bins (size 2mm). Randoms and scatters were simulated by uniform and blurred (convolution of true projections with a Gaussian kernel of 10 bins standard deviation) sinograms respectively, with a scatter fraction of 20% and a randoms fraction of 20%. Three different scaling factors were applied to this projected data in order to generate three different mean count levels in the sinogram, prior to the introduction of Poisson noise into each sinogram bin. These scaling factors were chosen so as to obtain datasets containing ${3.5\times 10^{5}}$, ${3.5\times 10^{6}}$ and ${3.5\times 10^{7}}$ mean total counts in each 2D sinogram. These mean total counts corresponded to low, mid, and high count acquisitions. Hence a broad range of count levels were considered, covering the case where substantial regularisation is required, through to the case where only a very low level of regularisation is needed.

#### 2) Reconstruction Methods:

**Fig. 2.**
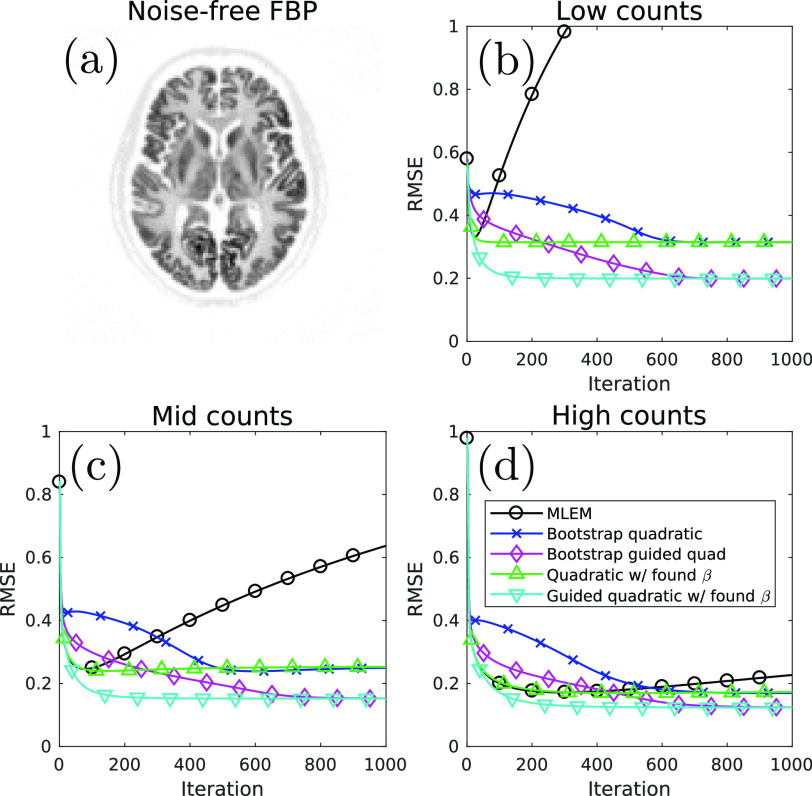
(a) The noise-, scatter-, and randoms- free non-resolution degraded FBP reference image used for RMSE calculation. (b-d) RMSE as a function of iteration for the three count levels for conventional unregularised MLEM image reconstruction, as well as for the bootstrap-optimised implementations of MAPEM using the unweighted quadratic penalty and the guided quadratic penalty. For each penalty, $\lambda ^{({0}{)}}$ and ${N}$ (see (10)) were held constant for *all* count levels, indicating that the proposed methodology is robust over a wide range of noise levels. RMSE is also shown for standard MAPEM reconstructions using the $\beta $ values provided by the proposed method, demonstrating that the method is converging to the fixed-$\beta $ solution at high iteration numbers.

Two regularisation functions were considered in the 2D simulation study: an unweighted quadratic penalty and a weighted quadratic penalty guided through use of side information (hereafter referred to as the guided quadratic penalty). Both penalties used $5\times 5$ neighbourhoods. The unweighted quadratic penalty was implemented as a weighted quadratic penalty with all weights $w_{jl}$ set to 1. To calculate weights for the guided quadratic penalty we used the Bowsher method, in which the $B$ most similar neighbouring pixels for each pixel in the guiding image are found, and the corresponding weights $w_{jl}$ are set to 1, with all other $w_{jl} = 0$ [26]–[28]. Similarity was based on the squared difference between pixel values, and the $B=10$ most similar pairings were kept per pixel, with similarities calculated from a noise-free, scatter- and randoms-free, non-resolution-degraded filtered backprojection (FBP) reconstruction as the guiding image (Figure 2(a)) (corresponding to a case where reliable high-quality side information is available). Note that with these definitions for $w_{jl}$, the two penalties have differing intrinsic scales since $\sum _{j} w_{jl} = 25$ for the unweighted quadratic penalty and $\sum _{j} w_{jl} = B = 10$ for the guided quadratic penalty. While this is expected to affect the magnitude of the $\beta $ values, it is not expected to affect the proposed method which is designed to automatically scale $\beta $.

There are two values that need to be set for the bootstrap-optimised framework described in this work: the cooling initialisation parameter $\lambda ^{(0)}$ and the smoothing cooling constant $N$. We found that $N=100$ iterations and $\lambda ^{(0)} = 1000$ gave good results in this work, although the proposed method was observed to perform well for any suitably large values of these parameters at the cost of increased reconstruction time, as demonstrated later. Furthermore, a mask was defined in which the image-space EMOF was optimised, to ignore surrounding empty regions when optimising $\beta $ values. This mask was calculated by segmenting the FBP reconstruction of the data as shown in Figure 2(a) into ‘head’ and ‘background’ regions, using a single threshold followed by morphological operations to fill any remaining holes. The 1D EMOF optimisation in (20) was performed with the MATLAB constrained optimisation function fmincon (using the documented interior point algorithm with scaled objective function and constraints). The analytic derivative of (20) was also provided to improve robustness, the Hessian was approximated by finite-difference methods, and the conjugate gradient method was used to solve the subproblems.

Each simulated dataset was reconstructed using conventional unregularised MLEM, the bootstrap-optimised unweighted quadratic penalty, the bootstrap-optimised guided quadratic penalty, and standard regularised reconstructions (with fixed $\beta $ values). Resolution modelling using a 3mm FWHM Gaussian kernel was included, and all reconstructions were run for 1000 iterations to ensure convergence of the regularised methods. Reconstructed images had a pixel size of $2\times {2}{\text {mm}^{2}}$. It is important to note that with the cooling scheme used, a fixed final $\beta $ is found for each bootstrap-optimised regularised method, resulting in a conventional convergent regularised reconstruction in every aspect except for how the regularisation strength was set.

The bootstrap replicate of the noisy measured data, required by the proposed method, was found through the method previously described in section III.

#### 3) Image Evaluation:

The reconstructed images were evaluated over $S = 10$ noise realisations, found to be sufficient for the below metrics which are evaluated over the large masked region of the images, defined as $\Omega $. The average root mean square error (RMSE) was calculated by

\begin{equation*} \textrm {RMSE} = \sqrt {\textrm {SD}^{2} + \textrm {Bias}^{2}} \tag{21}\end{equation*}

with the bias given by

\begin{equation*} \textrm {Bias} = \sqrt {\frac {\sum \limits _{j\in \Omega }\left ({\bar {\theta }_{j}^{(k)} - \theta _{j}^{\textrm {Ref}}}\right)^{2}}{\sum \limits _{j\in \Omega }\left ({\theta _{j}^{\textrm {Ref}}}\right)^{2}}} \tag{22}\end{equation*}

and the standard deviation (SD) given by

\begin{equation*} \textrm {SD} = \sqrt {\frac {1}{S} \frac {\sum \limits _{s=1}^{S}\sum \limits _{j\in \Omega }\left ({\bar {\theta }_{j}^{(k)}-\theta _{j}^{(s,k)}}\right)^{2}}{\sum \limits _{j\in \Omega }\left ({\theta _{j}^{\textrm {Ref}}}\right)^{2}}}. \tag{23}\end{equation*}

Here, $\bar {\theta }_{j}^{(k)}$ is the mean reconstructed value for voxel $j$, found by taking the average of the $S$ noisy realisations and $\boldsymbol {\theta }^{\textrm {Ref}}$ is a reference image for error calculation. The noise-free, scatter and randoms free, non-resolution-degraded FBP reconstruction was used as the reference image in all cases (Figure 2(a)).

### B. Results

Figure 2(b-d) shows the reconstruction RMSE as a function of iteration for conventional unregularised MLEM reconstructions at each noise level, alongside the corresponding results for the two bootstrap-optimised regularisation methods. While MLEM RMSE levels increase at high iterations due to the progressive fitting of noise in the data, the bootstrap-optimised regularised methods stabilise to a fixed level of reconstruction error. Furthermore, these error levels are either comparable to, or superior to, the minimum RMSE across all iterations achieved by MLEM. Specifically, the unguided quadratic penalty provides error levels similar to the optimum MLEM error levels and the guided quadratic penalty outperforms even the optimum MLEM reconstruction, particularly in the low-count case. This occurs due to the guidance providing additional information to the reconstruction that is not contained in the data, allowing a performance improvement compared to using the data alone with assumptions of isotropic smoothness. Figure 2(b-d) also shows RMSE as a function of iteration for the case where a standard MAPEM reconstruction is performed using the final value of $\beta $ provided by the proposed method (i.e. $\beta = \beta _{\textrm {cool}}^{\textrm {(end)}}$). For all noise levels the RMSE at 1000 iterations using bootstrap optimisation is the same as that obtained by using the fixed-$\beta $ approach, demonstrating that the proposed method converged successfully. This reinforces the validity of the final-iteration image provided by the proposed method, without the need to restart the reconstruction with the obtained $\beta $ value.

**Fig. 3.**
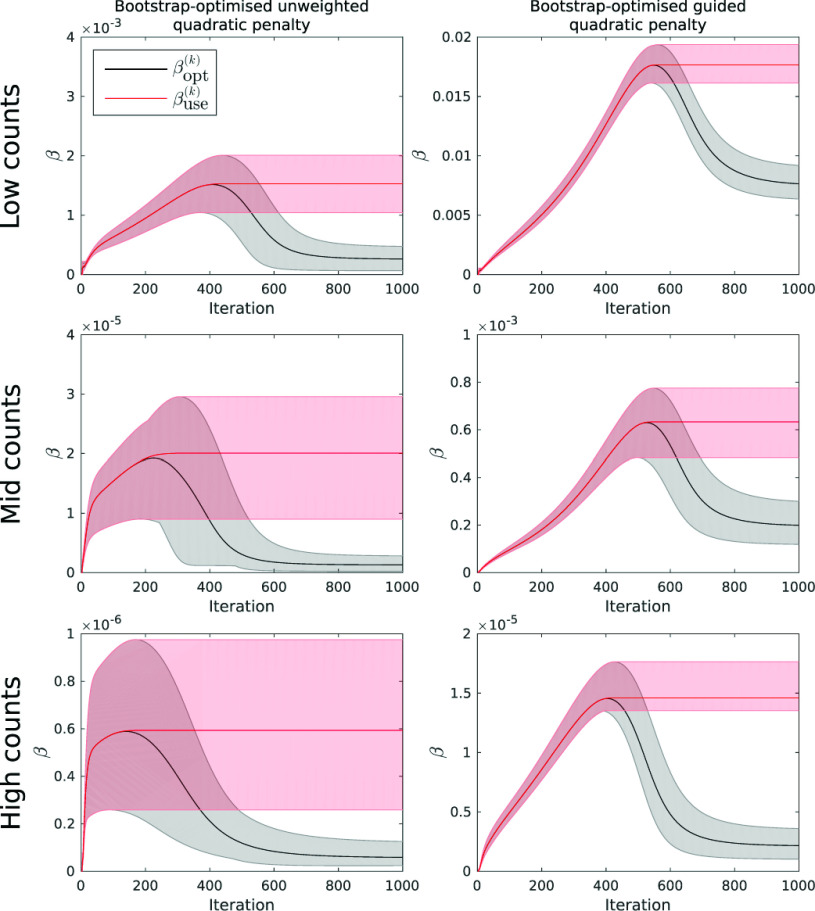
Progression of $\beta _{\textrm {opt}}$ and $\beta _{\textrm {use}}$ as a function of iteration for the bootstrap-optimised reconstructions. $\beta _{\textrm {cool}}$ is omitted for clarity, and by definition approaches $\beta _{\textrm {use}}$ at high iterations. Shaded error bars show the range for each value (over 10 independent noise realisations), and solid lines show the mean values. Note how $\beta _{\textrm {opt}}$ peaks before dropping to settle at a low value at higher iterations, in accordance with the theory (Section II.B).

Figure 3 shows the values of $\beta _{\textrm {opt}}$ and $\beta _{\textrm {use}}$ as a function of iteration for each penalty and count level. As well as the mean values (solid lines), the range of values found across the 10 noisy realisations are also displayed. The progression of the optimised $\beta $ values exhibit a similar behaviour in all cases. As count levels increase, the bootstrap-optimised values of $\beta _{\textrm {opt}}$ correspondingly reduce, reflecting the fact that less regularisation is required for higher quality PET data.

**Fig. 4.**
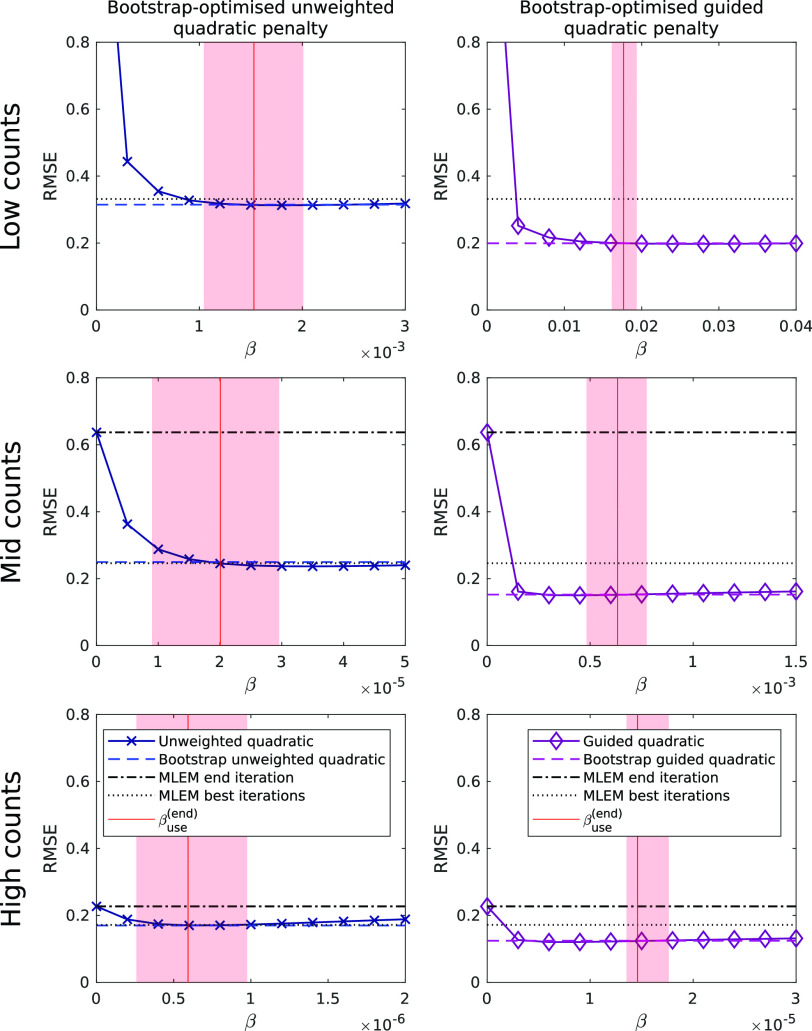
End-iteration RMSE as a function of $\beta $ for each penalty function, with the bootstrap-optimised RMSE values shown for comparison. MLEM RMSE values at both the end iteration (dot-dashed lines) and the best iteration (dotted line) are also shown. Note the different $\beta $ values required for each penalty. The bootstrap-optimised reconstructions achieve reconstruction error levels comparable to a grid search for all noise levels considered, but crucially, unlike a grid search, without knowledge of the ground truth and with use of the very same fixed values of $\lambda ^{({0}{)}}$ and ${N}$ for all cases.

Figure 4 shows RMSE as a function of systematically-chosen $\beta $ values for conventional regularised reconstructions. The RMSE for MLEM is also shown, for both the end iteration and the optimum iteration number (where lowest RMSE is found), and also the RMSE obtained using the bootstrap-optimised reconstruction methods. For each method at each noise level the bootstrap-optimised reconstruction provides error levels comparable to the optimum conventional regularised reconstructions. The range and mean of the bootstrap-optimised $\beta $ values $\beta _{\textrm {use}}^{(\textrm {end})}$ over the 10 noisy realisations of $\boldsymbol {m}$ are also shown for comparison.

**Fig. 5.**
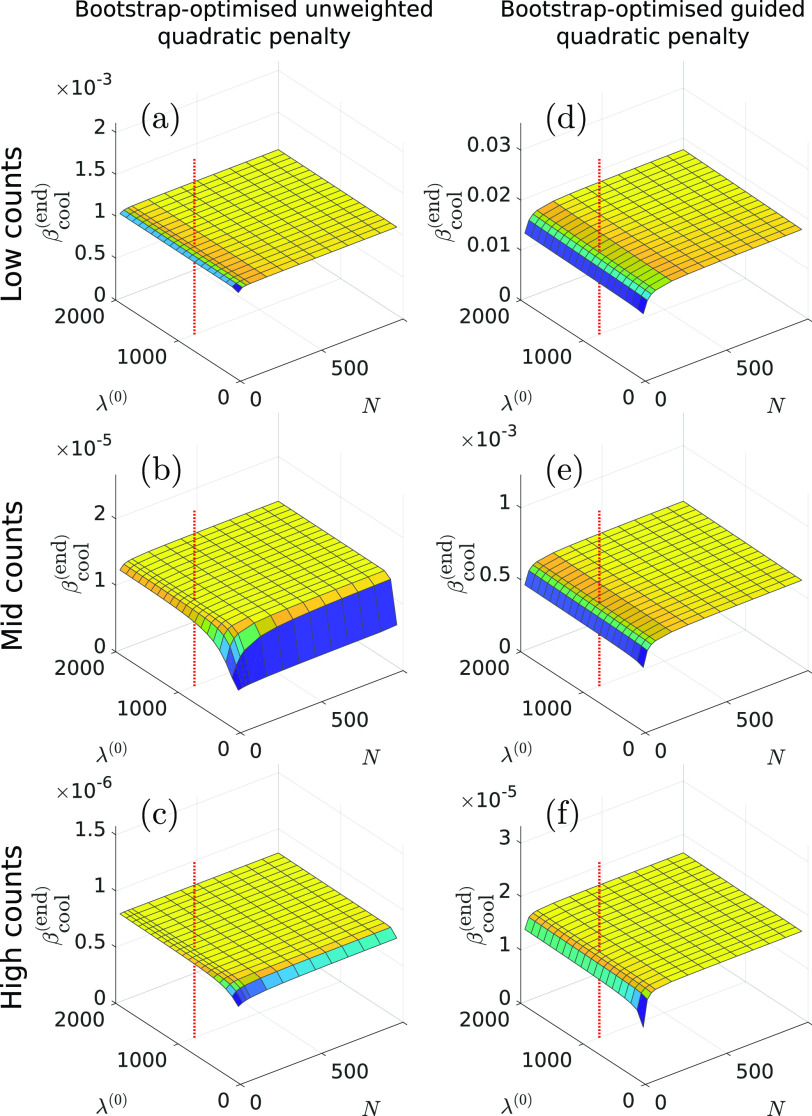
Final $\beta $ values provided by the proposed bootstrap-optimised reconstruction method ($\beta _{\textrm {cool}}^{({\text {end}}{)}}$) vs ${N}$ and $\lambda ^{({0}{)}}$. For sufficiently high values of ${N}$ and $\lambda ^{({0}{)}}$ the endpoint $\beta $-value is approximately constant. Vertical dashed lines show the values used in Figures 2–4; these lie within the approximately constant regions on the graphs, showing that these values are suitable for both penalty functions and all tested noise levels.

To verify the use of one-off fixed values for $N$ and $\lambda ^{(0)}$ irrespective of noise level, sinograms at the three different noise levels were reconstructed with a range of values for $N$ and $\lambda ^{(0)}$, and the final $\beta _{\textrm {cool}}^{(\textrm {end})}$ recorded (Figure 5). Just the one same bootstrap realisation for a given noise level was used for each reconstruction, and the number of iterations was set to $10N$ to ensure stabilisation of the found $\beta $ values. The results demonstrate a plateau in the $\beta _{\textrm {cool}}^{(\textrm {end})}$ values with sufficiently high $N$ and $\lambda ^{(0)}$, although with a slight tendency to higher $\beta $ values at higher values of $N$ and $\lambda ^{(0)}$. Nonetheless, it can be seen that the fixed values of $N$ and $\lambda ^{(0)}$ used previously (100 iterations and 1000 respectively, shown by dashed vertical lines in Figure 5) were high enough to provide $\beta $ values close to the maxima in all cases.

**Fig. 6.**
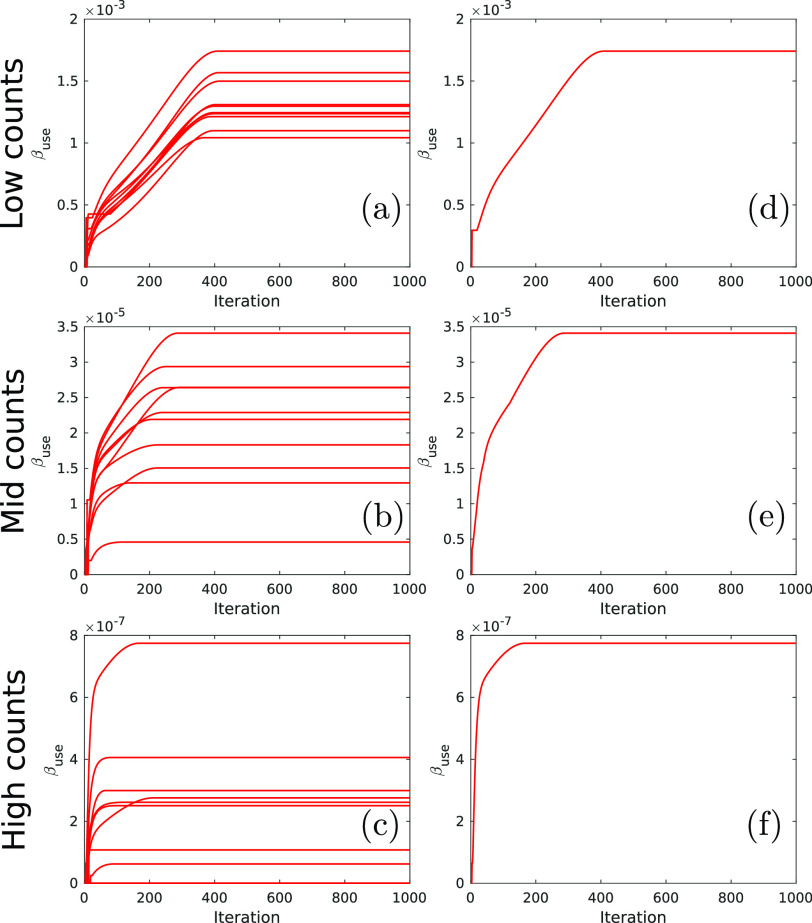
(a)-(c) Development of $\beta $ for 10 different reconstructions from a same single noisy dataset, where in each case a different bootstrap sample was used (each line is a single reconstruction). In the high count case, (c), some bootstrap samples result in a zero value of $\beta $, which is potentially problematic. (d)-(f) By including the 10 bootstraps in a single reconstruction and selecting, for example, the largest $\beta $ value found from all the bootstraps, the possibility of finding a zero $\beta $ for the high count case is avoided.

As only one single bootstrapped dataset $\boldsymbol {m}_{\textrm {boot}}$ has been used for computational efficiency, robustness of the method for different bootstrapped samples was investigated. We ran 10 reconstructions for a single identical noisy realisation of $\boldsymbol {m}$ using the unweighted quadratic penalty, with just the random seed for bootstrap generation changed in each of the 10 runs. All 3 count levels were considered. For the high count case the results in Figure 6 show that there is in fact potential to have bootstrap samples that deliver a zero value for $\beta _{\textrm {use}}$, for the case where extremely small values of $\beta $ are needed. For the lower count cases this issue is not encountered. To investigate how the possible pitfall of a zero $\beta _{\textrm {use}}$ value could be counteracted, we ran a single reconstruction with an extension of the proposed method: instead of calculating $\beta _{\textrm {opt}}^{(k)}$ based on a single $\boldsymbol {m}_{\textrm {boot}}$, we calculated 10 provisional $\beta _{\textrm {opt}}^{(k)}$ values based on 10 different bootstrap samples. We then chose the maximum as $\beta _{\textrm {opt}}^{(k)}$. This extension of the method to include multiple bootstraps in the same reconstruction results in endpoint $\beta $ values found for all count levels that are in very good agreement with the grid-searched optimal values (Figure 4), and counteracts the potential pitfall of a zero $\beta $ value.

**Fig. 7.**
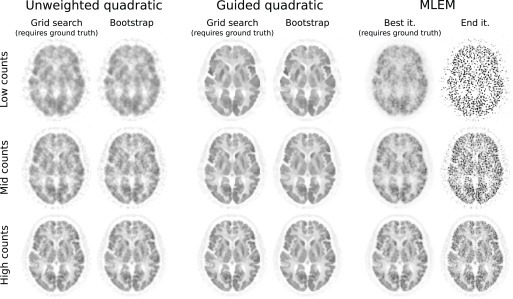
Example reconstructed images for each count level used in the 2D simulation studies. For each of the two quadratic penalties, images are shown at 1000 iterations using either the optimum $\beta $ value given by the ground truth grid search found in Figure 4, or the bootstrap-optimised reconstruction (which does not need the ground truth). MLEM reconstructions at the optimal iterations (as found in Figure 2), and the end-iteration MLEM reconstructions are also shown. The bootstrap-optimised regularisation method yields images qualitatively similar to the grid search results, reflecting the similar RMSE values observed in Figure 4, but entirely in absence of knowledge of the ground truth, as would be the case in practice.

In terms of visual assessment, Figure 7 shows that the bootstrap-optimised reconstructions are similar to the conventional regularised reconstructions when using the optimal $\beta $ values from Figure 4. Figure 7 also includes the MLEM reconstructions at optimal iterations, and end-iteration MLEM reconstructions, as useful reference points. It is again crucial to note that selection of these optimal reconstructions depends on knowing the ground truth, whereas the proposed method delivers comparable results in the absence of the ground truth.

## V. Application to Real Data

**Fig. 8.**
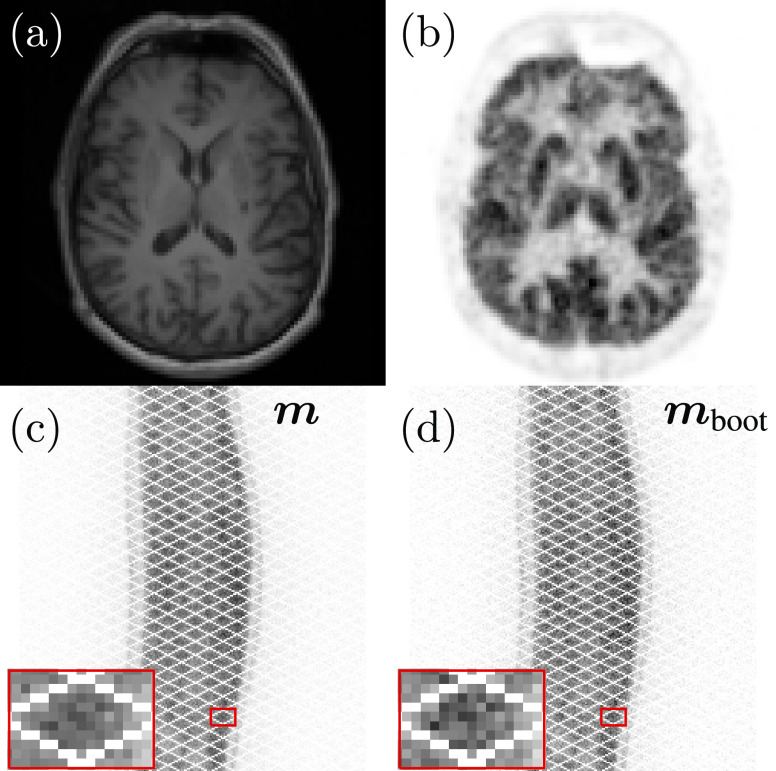
The patient PET-MR dataset used for the real data investigation. (a) The T1-weighted MR image resampled to the 3D PET image grid, (b) a slice of the 3D [^18^F]FDG image, reconstructed with 1000 iterations of MLEM followed by a 4mm FHWM Gaussian smooth, (c) a representative direct sinogram from the 3D [^18^F]FDG data acquisition ($\boldsymbol {m}$), and (d) an example bootstrapped replicate of the same direct sinogram ($\boldsymbol {m}_{\text {boot}}$). Red boxes in (c) and (d) show details of the sinograms for comparison.

Finally, the proposed bootstrap-optimised regularised reconstruction methodology was applied to a real [^18^F]FDG brain PET dataset (Figure 8). The data were acquired from an Alzheimer’s disease patient scanned using a Siemens Biograph mMR PET- magnetic resonance (MR) scanner (Siemens Healthcare, Erlangen, Germany). The patient was injected with 229.1MBq of [^18^F]FDG 70min prior to a 30min acquisition, resulting in a total of 563 million recorded prompt counts. PET attenuation maps were produced using the default ultrashort echo time MR sequence. Randoms and scatters were estimated from the list-mode data using the vendor-supplied e7-tools software (Siemens Healthcare, Knoxville, TN, USA). The dataset also included a simultaneously acquired T1-weighted MR image that can be used for anatomically-guided reconstruction [Figure 8(a)].

### A. Experimental Methods

The key benefit of the proposed method is its robustness to varying levels of statistical noise. Therefore, the original, full-counts patient dataset was used to generate additional datasets with reduced numbers of counts by selecting counts from the sinogram with a fixed probability, to obtain different noise levels for testing. For example, by retaining counts with a 10% probability, a 10%-counts Poisson-noisy dataset can be generated. Using this method, high, mid, and low counts datasets were produced, containing on average 100%, 10%, and 1% of the original counts. The scatters and randoms estimates were scaled accordingly rather than being recalculated at each noise level.

These real datasets were then reconstructed with 1000 iterations of the proposed method, using both the unweighted and guided (by Bowsher-weights) quadratic penalties. Both methods used a neighbourhood size of $3 \times 3 \times 3$ and the Bowsher weights were calculated from the T1-weighted MR image using the $B = 7$ most similar neighbours for each voxel. The values of $\lambda ^{(0)}$ and $N$ were 1000 and 50 iterations respectively. Compared to the 2D simulation case, $N$ was reduced since 3D reconstructions are known to converge more quickly than 2D reconstructions. The 1D EMOF optimisation (20) was performed using the interior point algorithm and also the ‘trust region reflective’ algorithm from the MATLAB function fmincon. The best $\beta $ value from the two solvers was used as $\beta _{\textrm {opt}}^{(k)}$. In this case each algorithm was also provided with the gradient of the EMOF (differentiating (20) with respect to $\beta $). The attenuation maps, which contain information about the object location, were used to define the mask in which the EMOF should be optimised. For the brain data used in these tests, the EMOF mask was calculated by performing a binary thresholding on the vendor-provided attenuation maps, followed by morphological operations to close and fill any remaining holes in the mask. Reconstructions included PSF-modelling with a 3mm FWHM Gaussian kernel, and the reconstructed voxel size was $2.08626 \times 2.08626 \times \,\,{2.03125}{\text {mm}^{3}}$.

For comparison purposes, MAPEM reconstructions with three fixed $\beta $ values were also performed for each penalty to demonstrate the range of penalty strengths required over the three count levels investigated. For the unweighted quadratic penalty these three $\beta $ values were 10, 1000 and 50000 and for the guided quadratic penalty they were 10, 1000 and 100000.

### B. Results

**Fig. 9.**
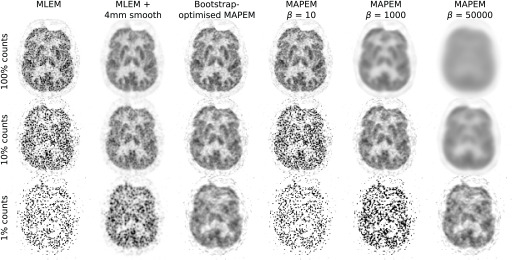
Real data PET image reconstructions using MLEM, 4mm-smoothed MLEM and the bootstrap-optimised unweighted quadratic penalty method. Standard unweighted quadratically-penalised MAPEM reconstructions using fixed $\beta $ values are also shown. As counts decrease, MLEM becomes noisy even with a (clinically typical) 4mm Gaussian smooth. Using standard MAPEM requires an appropriate counts-dependent selection of $\beta $. In contrast, the proposed bootstrap-optimised MAPEM provides good noise reduction at all count levels, without any specification of penalty strength. $\beta _{\textrm {cool}}^{({\text {end}}{)}}$ values for the bootstrap-optimised reconstructions were (from high to low counts) 18.4, 1028, and 49514.

**Fig. 10.**
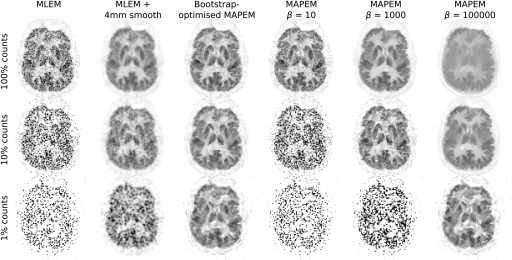
Real data PET image reconstructions using the bootstrap-optimised *guided* quadratic penalty method. As seen in Figure 9, the bootstrap-optimised MAPEM method automatically selects suitable $\beta $ values at each noise level. $\beta _{\textrm {cool}}^{({\text {end}}{)}}$ values for the bootstrap-optimised reconstructions were (from high to low counts) 52.6, 3371, and 111635.

The reconstructed real data images using the unweighted quadratic penalty are shown in Figure 9. As expected, noise in the MLEM reconstructions increases with decreasing counts, to the point where a standard 4mm smooth becomes insufficient at 1% counts. The MAPEM reconstructions with different $\beta $ values show the risks of using a fixed $\beta $ value in terms of variation of optimal penalty strength at different noise levels. For example, when using $\beta = 50000$ (which performs well at 1% counts) the 100% counts dataset is highly over-regularised, losing all detail completely. The bootstrap-optimised reconstruction method, however, adaptively determines the penalty strength on-the-fly during the iterative reconstruction and so can appropriately regularise the reconstructed images at any noise level. This leads to reduced regularisation at low noise levels (hence retaining detail), and increased regularisation at high noise levels (hence reducing potentially misleading details arising from noise). Similar results are observed when considering the guided quadratic penalty results (Figure 10), where again the penalty strength for the very low count case is appropriately amplified.

## VI. Discussion

This work proposes a general, unsupervised optimised regularised image reconstruction methodology applicable to many iterative methods, which automatically selects an appropriate value of the penalty strength $\beta $ using bootstrap methods to model the ensemble mean. The method can in principle be extended to select other hyperparameters related to regularisation. Figures 2 and 3 show that the proposed method, for the two implemented penalty functions, progresses towards fixed $\beta $ values, providing stable reconstructions at high iteration numbers. Provided a convergent MAPEM algorithmic framework is selected, then the final outcome of the proposed method is correspondingly a convergent regularised reconstruction method. In the current work, both a guided and also an unweighted quadratic penalty were used to demonstrate the methodology. While the proposed method performed very well in both cases, better RMSE values were obtained when using the guided quadratic penalty (particularly at lower SNR data), but of course such a penalty is wholly reliant on the availability of high-quality side information. Nonetheless, the use of bootstrap-optimised regularisation highlighted the benefit of high-quality side information, and furthermore since it provides optimised reconstructions for both penalties, it is easier to observe the lower reconstruction error obtained when side information is available and included (Figure 2). In this manner, bootstrap-optimised regularisation may serve as a research tool to allow a more objective comparison between penalties, allowing each regularisation to perform at or near its optimal point, potentially even assisting in choosing which penalty to use.

The reconstruction errors of the images were similar to those obtained by regularised reconstructions using an exhaustive grid search to find $\beta $ (Figure 4), which, however, also needed knowledge of the ground truth. The key benefit of the proposed methodology is that it operates on-the-fly, without supervision, to deliver precise levels of regularisation without an exhaustive search, and without knowledge of the ground truth.

The use of a single bootstrap replicate can lead to $\beta $ values of 0 when using very high-statistics PET data (which requires only very small values of $\beta $), as was observed in Figure 6(c). Robustness of the proposed method can be improved through use of multiple bootstrap replicates. Figure 6 demonstrated that the potential pitfall of a zero $\beta $ could thus be avoided by considering updates from multiple bootstrap replicates.

One possible present limitation of the methodology is the number of iterations involved (e.g. 1000 iterations were used here to ensure the endpoint $\beta $ value had settled and that the reconstruction had time to converge for that final $\beta $ value). Therefore future work could consider acceleration possibilities for the proposed method.

Another area of potential future work is to assess any possibility of object, scanner or ET-modality dependence of the initial over-regularisation and cooling scheme. The very strong over-regularisation and slow cooling scheme presently employed means that early iterations of the method should always be sufficiently over-regularised irrespective of the object, count level, scanner or ET modality. This could be verified by more extensive testing over a range of representative objects and ET modalities, potentially even with a view to reducing or automatically adapting the initial over-regularisation and cooling scheme, thereby reducing reconstruction times. However, the emphasis in the present work was to provide an approach that should prove robust to many different ET imaging scenarios.

When applied to an anecdotal real 3D PET dataset, the bootstrap-optimised methodology performed extremely well. The methodology adaptively selected $\beta $ values for three very different count levels, delivering images of good visual quality, whether using the unweighted quadratic penalty (Figure 9) or the guided quadratic penalty with Bowsher weights (Figure 10). In addition, there is scope to explore using the proposed bootstrap-based methodology to perform on-the-fly penalty-selection, for example selecting between incorporating guidance information or using an isotropic penalty, according to both the statistical quality of the PET data and the quality of the guidance information.

While the aim of this present work is to remove the reliance of regularisation methods on user-selected hyperparameters, it is nonetheless recognised that in some contexts it may be desirable to preserve a level of interactivity, e.g. to select the appearance or texture of residual noise in the images. Our proposed framework, in its present form, still leaves the choice of penalty (function $R$ in (1)) to the user. Hence there is still scope for interactivity by selecting a penalty term that models the desired end-point image characteristics. This could also include the use of spatially-adaptive regularisation to encourage spatially uniform noise levels or resolution [9]–[11]. The proposed bootstrap methodology would still find the appropriate level of regularisation for that choice of penalty.

The proposed bootstrap-optimised regularisation has potential application to many image reconstruction, restoration, and parameter estimation problems that suffer from high levels of noise. For instance, an application outside of medical imaging is reducing shot noise in natural or astronomical image processing. This has been the focus of previous work in automated hyperparameter estimation (e.g. [29]). Using the methodology proposed here to estimate and compensate for the intrinsic variance of the data on-the-fly could be a powerful tool in such contexts.

## VII. Conclusions

This work proposes the use of one or more bootstrap replicates of a noisy measured dataset to find an optimised level of regularisation for the reconstruction of that dataset. This is achieved by finding the regularisation hyperparameter $\beta $ that best fits an image update using the bootstrapped data to an image update using the original data. This value of $\beta $ corresponds to estimating the noise-free ensemble mean of numerous noisy updates. By choosing the maximum level of regularisation encountered during the entire sequence of iterative updates, the strongest necessary regularisation is selected for the final stages of the iterative reconstruction.

The results of 2D PET simulation studies showed great promise for the methodology, with reconstruction error levels and image appearance in good agreement with RMSE-optimal regularised reconstructions (found with an exhaustive hyperparameter search and knowledge of the ground truth). The method was then successfully applied to real 3D PET data, demonstrating ability to adaptively select appropriate $\beta $ values depending on the noise level, providing images of excellent visual quality.

In summary, a hyperparameter-free regularised image reconstruction methodology is proposed, simplifying the practical use of regularisation.
